# Artificial intelligence to detect *MYC* translocation in slides of diffuse large B-cell lymphoma

**DOI:** 10.1007/s00428-020-02931-4

**Published:** 2020-09-26

**Authors:** Zaneta Swiderska-Chadaj, Konnie M. Hebeda, Michiel van den Brand, Geert Litjens

**Affiliations:** 1 Department of Pathology, Radboud University Medical Center, Geert Grooteplein 10, P.O. Box 9101, 6500 HB Nijmegen, The Netherlands; 2 Faculty of Electrical Engineering, Warsaw University of Technology, Warsaw, Poland; 3 Pathology-DNA, Rijnstate Hospital, Arnhem, the Netherlands

**Keywords:** B-cell lymphoma, *MYC*, H&E, DLBCL, Deep Learning

## Abstract

In patients with suspected lymphoma, the tissue biopsy provides lymphoma confirmation, classification, and prognostic factors, including genetic changes. We developed a deep learning algorithm to detect MYC rearrangement in scanned histological slides of diffuse large B-cell lymphoma. The H&E-stained slides of 287 cases from 11 hospitals were used for training and evaluation. The overall sensitivity to detect MYC rearrangement was 0.93 and the specificity 0.52, showing that prediction of MYC translocation based on morphology alone was possible in 93% of MYC-rearranged cases. This would allow a simple and fast prescreening, saving approximately 34% of genetic tests with the current algorithm.

**Electronic supplementary material:**

The online version of this article (10.1007/s00428-020-02931-4) contains supplementary material, which is available to authorized users.

## Introduction

Diffuse large B-cell lymphoma (DLBCL) is the most common type of B-cell lymphoma and includes a diversity of not yet completely characterized clinico-pathological lymphomas [1]. A subgroup of 5–15% of DLBCL shows *MYC* oncogene rearrangement. Especially when combined with a *BCL2* and/or *BCL6* rearrangement (high-grade B-cell lymphoma with *MYC* and *BCL2* and/or *BCL6* rearrangement), these DLBCL have a poor outcome when treated with standard R-CHOP chemotherapy [2], and may require a different treatment [3]. Recent studies on molecularly defined DLBCL subgroups confirmed the poor prognosis of a MYC rearrangement [4]. Therefore, currently a genetic test for *MYC* rearrangement and, if positive, for *BCL2* and *BCL6*, is required for DLBCL patients for diagnosis, prognosis, and to guide therapy. Many pathological classifications are based on the fact that genetic changes in a tumor are reflected in aberrant transcription, changed protein expression, and often a characteristic morphology of the tumor cells or the tumor microenvironment. Several morphologic variants of DLBCL are recognized, but clinical relevance is not yet established [1]. We hypothesized that a trained computer algorithm will be able to predict *MYC* rearrangement from morphology on a standard hematoxylin and eosin (H&E)-stained slide of a DLBCL, thereby obviating molecular tests in the majority of cases that will be predicted to lack a MYC translocation.

## Methods

**Fig. 1:**
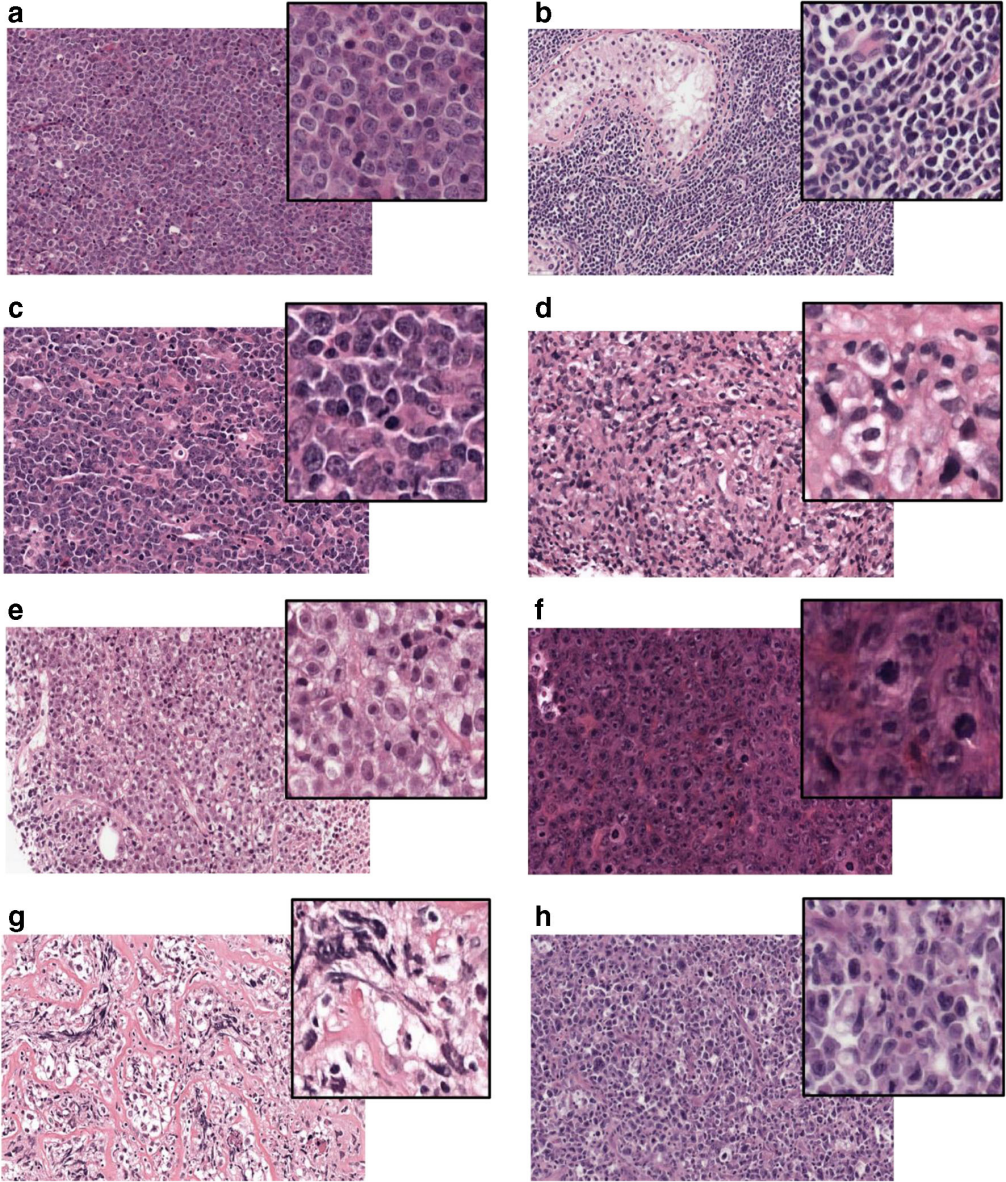
H&E slides of DLBCL with examples of morphological categories. **a** High-grade morphology (case 98); **b** high-grade morphology (case 78); **c** centroblastic morphology (case 260). **d** Centroblastic morphology (case 58). **e** immunoblastic morphology (case 134). **f** immunoblastic morphology (case 184). **g**. anaplastic morphology (case 255). **h** anaplastic morphology (case 44)

We collected an internal set of routinely stained H&E glass slides and *MYC* fluorescence in situ hybridization (FISH) test results of 245 patients that were diagnosed with DLBCL in 11 hospitals in the Netherlands, including our hospital (A) and 2 hospitals that contributed 23% (B) and 16% (C) of the cases. Slides for H&E and FISH were cut and stained at our department where the FISH was performed and interpreted between 2015 and 2019. Characteristics of the individual cases are presented in supplementary table 1. The morphology was extremely variable and sometimes very poor, but we tried to assign each case to one of the following categories: (1) high-grade morphology, including blastoid and Burkitt-like; (2) centroblastic, including large centrocytic, lobated, and elongated; (3) immunoblastic, consisting of at least 10% immunoblasts; and (4) anaplastic, including cases with very large, polymorphic, or Reed-Sternberg–like cells. Examples are given in Fig. 1. In addition, the presence of a strong inflammatory component (for example, histiocytes, eosinophils, or small lymphocytes) was noted, as was extensive diffuse or reticular fibrosis. In 31 cases, adequate morphological classification was not possible, mostly due to crush artifacts or bad fixation. The molecular subtypes germinal center B-cell (GC) or activated B-cell (non-GC) were recorded based on expression of CD10, BCL6, and MUM-1 according to the Hans-algorithm [5]. The EBV status of the DLBCL was noted as determined by in situ hybridization for EBER.

**Fig. 2:**
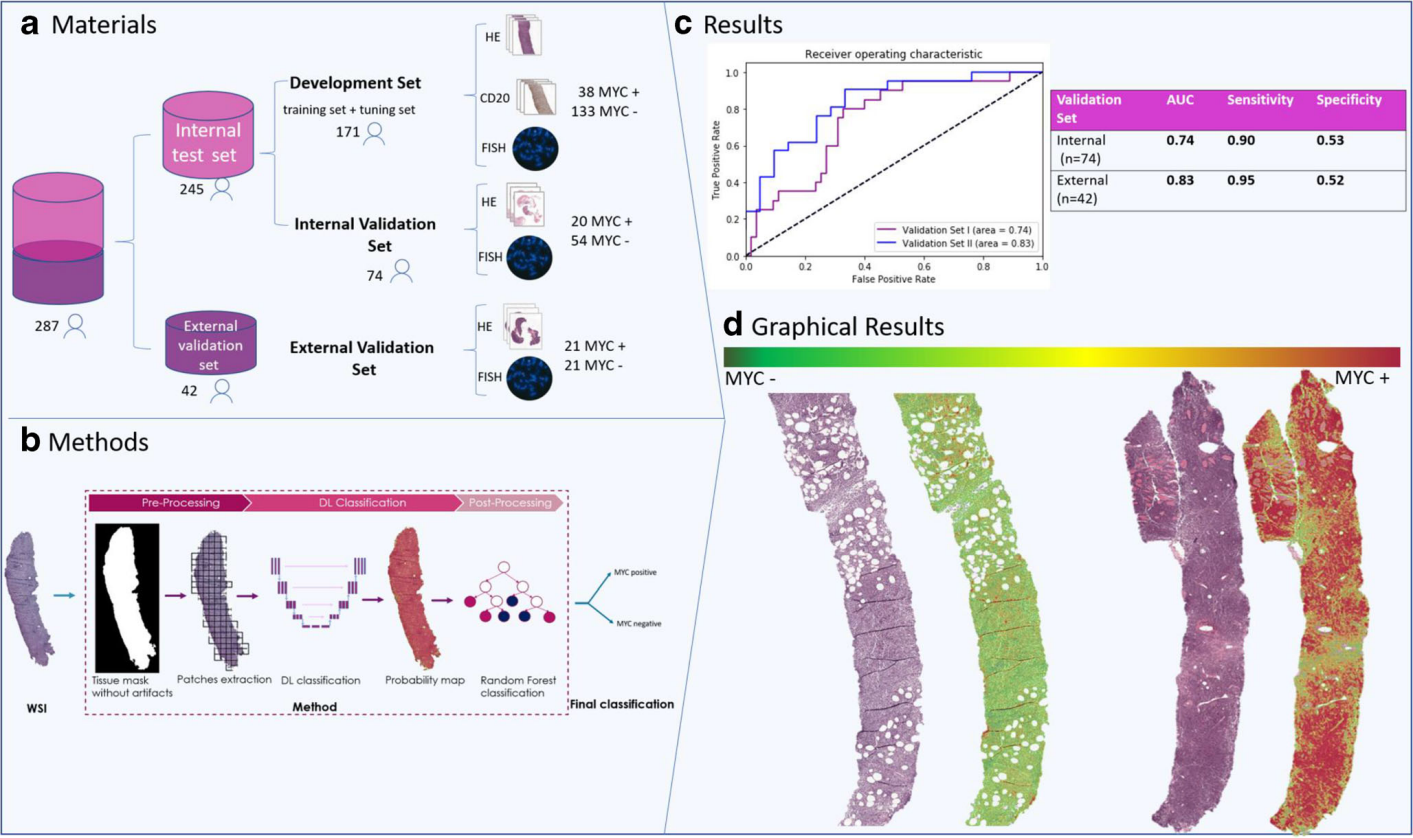
**a** Description of the patient groups that were used for training and validation of the algorithm. **b** Schematic steps that were used to create the final result of the algorithm. **c** The rate of false positive and negative results for the internal (red) and external (blue) validation set of DLBCL. **d** Two-needle biopsies of a *MYC*− (left) and *MYC*+ (right) DLBCL. The left image is a H&E-stained section, the corresponding digital image shows areas predicted to be *MYC*− in green and *MYC*+ in red.

The set was digitalized and the whole-slide images (WSI) were randomly divided into (a) training set consisting of 140 WSIs (31 with MYC rearrangement (MYC+) and 109 WSI without MYC rearrangement (MYC−)), to develop a machine learning algorithm; (b) a tuning set of 31 WSI (9 MYC+, 22 MYC−) to optimize a method development; and (c) an internal validation set consisting of 74 WSIs (20 MYC+, 54 MYC−) to evaluate the performance of the algorithm (Fig. 2a). For cases in the training and tuning set, we additionally used the diagnostic CD20 immunohistochemically stained slides (IHC) that were available in our archive to define the tumor area. Tumor areas and artifacts were digitally annotated by medical students, trained for this task under supervision of a pathologist (K.H.). All experiments were conducted in accordance with the Declaration of Helsinki and according to the Dutch “Code of Conduct” for responsible use.

The trained algorithm was also applied to an additional external validation set that consisted of 42 H&E slides of DLBCL from the archive of a participating hospital (Hospital C, Rijnstate hospital, Arnhem), with an equal proportion of MYC-rearranged and non-MYC-rearranged cases (21 each). This validation set was used to evaluate the performance of the algorithm on tissue that was processed and stained in a different hospital. All slides were digitalized using a Pannoramic 250 Flash II scanner (3DHistech, Hungary), resulting in WSIs with a pixel size of 0.24 μm (objective magnification × 20).

We applied a deep learning and classical machine learning approach, where the deep learning neural network (U-Net [6]) was trained using small areas from annotated WSIs. For each pixel, it gave a probability for the presence of a *MYC* rearrangement. After analysis of the whole slide, a *MYC* translocation likelihood map was created (Fig. 2d). Finally, the WSI was classified as MYC+ or MYC−, based on a Random Forest (RF) classification.

A pre-processing step was applied, where artifacts (tissue folds, ink, staining artifact) were eliminated from the analyzed areas by a different deep learning model. In this study, we applied a modified version of the deep learning method described previously [7]. Details are presented in the supplementary materials. The method was evaluated as a binary whole-slide classification task (*MYC*+ or *MYC*−) using receiver-operating characteristic (ROC) analysis for the internal and external validation sets.

## Results and discussion

The algorithm was able to identify *MYC*+ DLBCL areas within WSI of tissue biopsies and resections (Fig. 2d). We were able to detect 93% of the *MYC* rearranged DLBCL based only on the evaluation of an H&E-stained slide (sensitivity 0.90/0.95 and specificity 0.52/0.53 for the internal and external validation sets, respectively; Fig. 2c). This was unrelated to the tissue type or organ with DLBCL infiltration, including bones, soft tissues, intestines, and lungs (see supplementary Table 1 and Table 2). The variability in processing of the tissue, including fixation and if necessary decalcification, due to the acquisition from 11 different hospitals did not influence the results. The 3 false negative (FN) cases within the validation and test sets of 116 cases, were lymph nodes from 3 different hospitals. For hospitals A, B, and C with most tested cases, the false positive (FP) rate ranged from 17 to 33%, compared to a total FP rate of 31% (36 FP of 116 cases of the internal and external validation set). The type of tissue (lymph node or extranodal) did not clearly influence the FP rate (42% and 38%, respectively). Approximately half of FP and *MYC* negative cases had a GC phenotype (50% and 48%, respectively), which was less than in the *MYC* positive group (85%). High-grade, centroblastic, immunoblastic, and anaplastic morphology were seen in 24%, 46%, 12%, and 5% of the 41 MYC positive cases; 6%, 64%, 19%, and 19 % of the 36 FP cases; and 7%, 64%, 11%, and 20% of the 75 MYC negative cases. These data show an enrichment of GC phenotype and high-grade morphology in the MYC positive group, compared to the MYC negative cases. This is expected to be caused by the presence of “double-hit lymphoma” with an additional BCL6 or BCL2 rearrangement in the MYC positive group. EBV status was not known for all cases, but 2 of the 5 known EBV+ DLBCL were FP and one was MYC+. Because of this low number no conclusions can be drawn.

Our results show that clinical application of the algorithm could allow a very fast and cheap prescreening that can direct the application of genetic tests for *MYC* rearrangement. Attempts to prescreen for *MYC* rearranged cases using IHC for c-*myc* protein expression of the lymphoma cells show a lower sensitivity (0.88 for IHC versus 0.93 for the algorithm) and a comparable specificity (0.52) [8]. Prescreening with the algorithm would have saved 34% of the FISH tests if only positive predicted cases (41 *MYC*+ and 36 FP of 116 cases = 66%) are confirmed by FISH, as is common practice in many laboratories.

This is the first proof-of-principle that a conventional H&E slide of DLBCL harbors morphologic data that can predict the presence of genetic changes. Larger studies will be necessary to improve the results and to investigate whether DLBCL with different molecular background, as defined recently by several groups, can also be detected [4]. The ultimate question will be if it is possible to train algorithms to predict the response of a DLBCL to a specific treatment without knowledge of the molecular background of the tumor. To investigate this future perspective, large studies with extensive datasets are needed. Possibly the information of the H&E slides alone will not be sufficient and has to be completed with other clinical or biological data.

## Electronic supplementary material

ESM 1 (DOCX 2.52 mb)

ESM 2 (PDF 559 kb)
