# Supplementary material for: Artificial intelligence to detect MYC translocation in slides of diffuse large B-cell lymphoma
Source: Virchows Arch. 2020 Sep 26;479(3):617–21. doi: 10.1007/s00428-020-02931-4 (PMC8448690; doi:10.1007/s00428-020-02931-4)
Supplement: Supplementary file 1 — (DOCX 2.52 mb) [file 428_2020_2931_MOESM1_ESM.docx]

**SUPLEMENTARY MATERIALS:**

**MATERIALS**

Table 1. Summary of details of the individual DLBCL cases that were used for training, tuning and validation.

| Feature | value | SET | | | | |
| --- | --- | --- | --- | --- | --- | --- |
|  |  | Training  n=140 | Tuning  n=31 | Internal validation  n=74 | External validation  n=42 |  |
| MYC Fish | Positive:  Negative: | 31 (22%)  109 (78%) | 9 (29%)  22 (71%) | 20 (27%)  54 (73%) | 21 (50%)  21 (50%) |  |
| High-grade morphology | Yes:  No:  NE: | 17 (12%)  103 (74%)  20 (14%) | 3 (10%)  26 (84%)  2 (6%) | 8 (11%)  62 (84%)  4 (5%) | 7 (17%)  30 (71%)  5 (12%) |  |
| Centro-blastic | Yes:  No: | 87 (62%)  53 (38%) | 18 (58%)  13 (42%) | 42 (57%)  32 (43%) | 25 (60%)  17 (40%) |  |
| Immuno-blastic | Yes:  No: | 21 (15%)  119 (85%) | 6 (19%)  15 (81%) | 9 (12%)  65 (88%) | 4 (10%)  38 (90%) |  |
| Anaplastic | Yes:  No: | 10 (7%)  130 (93%) | 2 (6%)  29 (94%) | 14 (19%)  60 (81%) | 3 (7%)  39 (93%) |  |
| Morphology NE* | Yes:  No: | 21 (15%)  119 (85%) | 2 (6%)  29 (94%) | 4 (5%)  70 (95%) | 5 (12%)  37 (88%) |  |
| Inflammation | Yes:  No: | 4 (3%)  136 (97%) | -  31 (100%) | 5 (7%)  69 (93%) | 2 (5%)  40 (95%) |  |
| Fibrosis | Yes:  No: | 30 (21%)  110 (79%) | 2 (6%)  29 (94%) | 17 (23%)  57 (77%) | 7 (17%)  35 (83%) |  |
| IHC | GC:  Non-GC:  Unknown:  CD10neg: | 73 (52%)  32 (23%)  10 (7%)  25 (18%) | 18 (58%)  8 (26%)  4 (13%)  1 (3%) | 42 (57%)  21 (28%)  8 (11%)  3 (4%) | 29 (69%)  9 (21%)  2 (5%)  2 (5%) |  |
| EBER | Positive Negative:  Unknown: | 6 (4%)  71 (51%)  63 (45%) | -  18(58%)  13(42%) | 4 (5%)  45 (61%)  25 (34%) | 1 (2%)  31 (74%)  10 (24%) |  |
| Site | Lymph node  Extranodal | 36 (26%)  104 (74%) | 5 (16%)  26 (84%) | 22 (30%)  52 (70%) | 22 (52%)  20 (48%) |  |
| Hospital | A:  B:  C:  D: | 109 (78%)  19 (14%)  1 (1%)  11(8%) | 19 (61%)  7 (23%)  3 (10%)  2 (6%) | 17 (23%)  39 (53%)  1 (1%)  17 (23%) | -  -  42 (100%)  - |  |

*where: GC- germinal center; NE- not evaluable; Non-GB- non- germinal center; Hospitals: A-Radboud hospital, B-CWZ, C- Rijnstate hospital, D-other hospitals.

Table 2 with detailed information for each case is attached as a separate file due to the large size of the table.

**METHOD DESCRIPTION:**

The used method is based on a tiles semantic segmentation approach by convolutional neural network and a rule-based slide-level classification [Swiderska-Chadaj, 2020], where we can distinguish four main steps: (I) pre-processing, (II) slide normalization applied only for validation slides, (III) deep learning (DL) classification, and (III) postprocessing. Figure 3 presents the schema of proposed method.


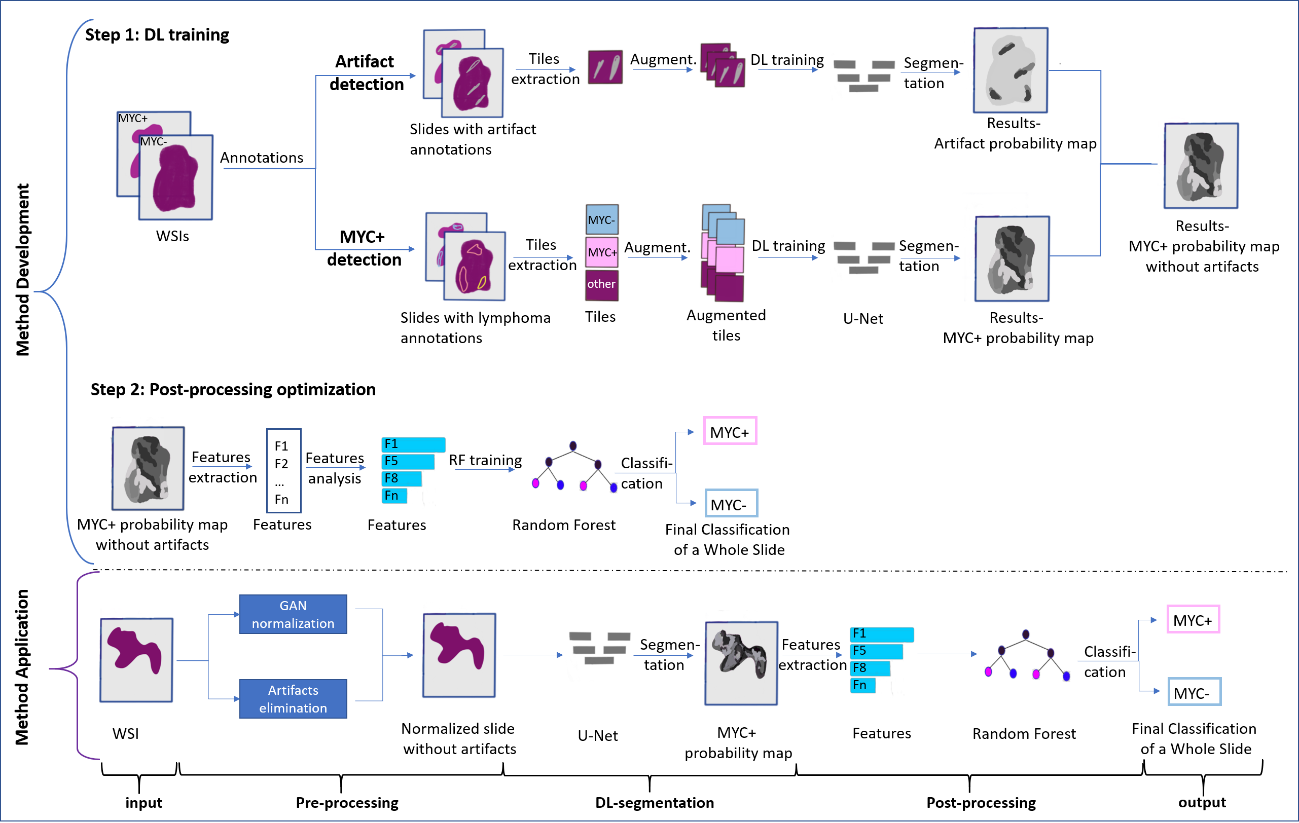


Figure 1. The scheme of the applied training method.

**Slide normalization:** The specimens prepared in the various labs are characterized by significant color variability. Depending on local protocols the tissue on the slides can be more pink or violet. This variety can affect DL classification. Slides in the development dataset were not normalized to keep color. In order to increase method robustness, we applied a slide normalization procedure for all validation slides. Colors in the validation slide were normalized to the colors in the development dataset by applying the GAN normalization technique [de Bel, 2019].

**Pre-Processing (artifact detection):** The pre-processing step includes tissue segmentation [Bándi,2019] and artifact detection and eliminations. H&E stained slides show a large variety of artifacts that can impair deep learning classification. The artifact elimination was performed as a separate step by a trained DL model and results in an artifact mask. The U-Net model [Ronneberger, 2015] was trained to detect artifacts based on artifact annotations prepared for the development set.

**DL segmentation:** The presented task is formulated as a whole-slide level classification. Due to the memory constraints and large size of whole slide images (2-4GB for x20), it is impossible to use a whole slide as a network input. Therefor we decided to use small tiles (Figure 4) and a slide windowing approach.


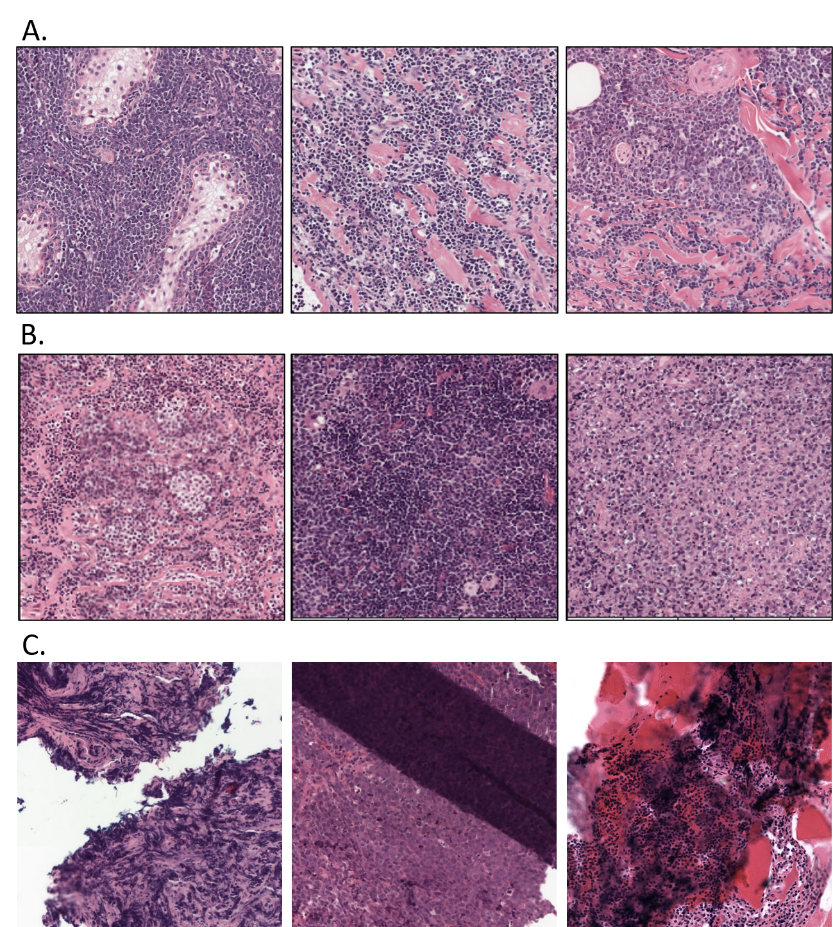


Figure 2. Example of tiles, where: A- tiles extracted from MYC positive cases; B-tiles extracted from MYC negative cases; C- tiles with artifacts.

The size of the tiles and the used resolution was discussed with the pathologists, in order to include sufficient tissue areas and cell details at the same time (if using tile with a high resolution we can see single cells but information about spatial patterns will be lost, and when using too small tile resolution each cell is represented only by few pixels causing loss of important information about cells). In the literature, two main deep learning strategies are available: patch classification using networks such as FCNN, ResNet, EfficientNet, or a semantic segmentation approach using networks such as U-Net, SegNet, DenseNetFCN. In our studies, we investigated both strategies (classification by FCN and segmentation by U-Net). Since in the preliminary studies better results were obtained with the semantic segmentation approach, we further developed this strategy.

As a result, the MYC classification was performed based on a semantic segmentation (pixel classification) by U-Net [Ronneberger,2015]. The training dataset was extracted from annotated tumor areas on H&E stained digitalized slides, where the *MYC* rearrangement status was evaluated on a whole slide level based on the FISH study. A single tile had a size of 512x512 pixels and pixel size 1um (magnification 5x). Tiles were extracted automatically from annotated areas, where each tile is fully included in annotated areas. Due to the lack of ground truth labels on a pixel level and of sparse annotations, for each extracted tile a target map including a single class (MYC positive or MYC negative) was created. The prepared target maps were used in the learning procedure of the deep learning model. Extensive data augmentation [Tellez 2019], based on a modification of brightness, contrast, saturation, and rotation, as well as additive Gaussian noise and Gaussian blur augmentation was applied to increase the number of training tiles. In order to improve our method, we adapted the original U-Net architecture by increasing network depth to 5 levels and adding spatial dropout layers with factor 0.25 between convolutional layers, with the aim of reducing overfitting. The U-Net model was trained with learning rate 0.0005, Adam optimizer and categorical cross entropy loss function by 500 epochs with batch size 200 and mini-batch size 1. The validation loss was monitored during the training, and the best model was used in the final classification. After training the network was used to generate an *MYC* rearrangement likelihood map for the H&E slides. The details about the training strategy are presented in Swiderska-Chadaj et al. [Swiderska-Chadaj,2020].

**Post-processing**:

In order to get the final classification on a whole slide level, it is necessary to build a decision-making system. If the DL network would correctly predict every piece of tissue as MYC positive or negative, a simple average or max pooling would result in a good slide-level likelihood. However, there are several challenges that complicate this. For example, the artifacts detection network is not 100% accurate, causing it to sometimes miss an artifact which can cause a significant amount of high likelihood false positives. By opting for the random forest (RF) approach, we make the whole pipeline more robust to these outlier classification errors from the deep learning pipeline, resulting in higher performance. The final decision that is based on several features is more robust on false positive detections caused by slide variability of the slide (including staining intensity, color, and scanning variability) or tissue features (different organs) than a simple evaluation of a predication map by thresholding. After DL classification a false positive area detection can occur on a slide (see figure 3), thus a more sophisticated approach than simple thresholding is necessary. The final slide classification as *MYC* positive or *MYC* negative was performed in the post-processing step, based on Random Forest (RF) feature classification. The Minimum Redundancy Maximum Relevance (mRMR)[ Radovic, 2017] method was applied to selected classification features. As a result, four cumulative histogram features showing the highest correlation with the class (output) and the least correlation between themselves were selected, namely: hist_03, hist_02, hist_04, hist_08. The RF was optimized using the development set.


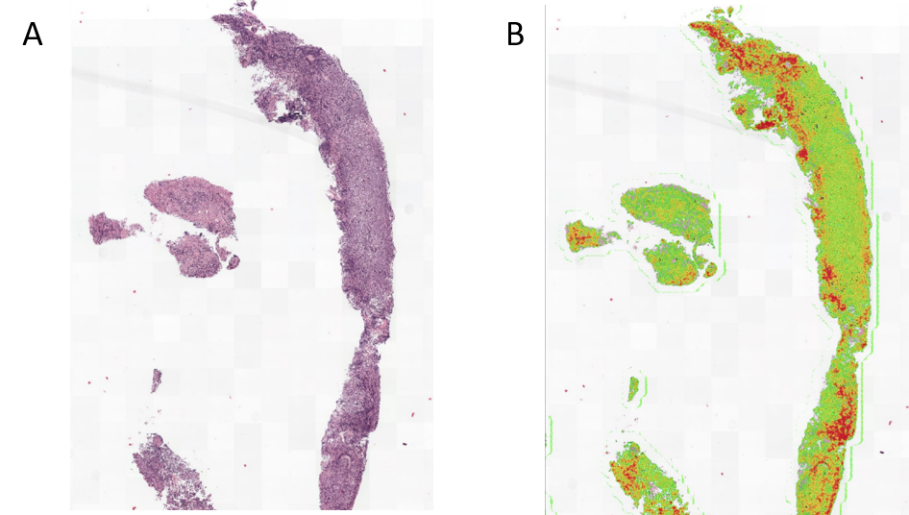


Figure 3. Example of false positive detections. A. H&E slide, B. MYC + probability map, red areas present false positive detections, results for case 47. An application of the post-processing step allows for correct whole-slide classification.

**Detailed results**:

Table 3. Results achieved for the internal and external validation of the algorithm detecting *MYC* rearrangement on an H&E WSI of DLBCL.

| Validation Set | AUC* | Sensitivity | Specificity | False Negative/ Positive | False Positive/ Negative |
| --- | --- | --- | --- | --- | --- |
| Combined | 0.77 | 0.93 | 0.52 | 3/41 | 36/75 |
| Internal validation set (n=76) | 0.74 | 0.90 | 0.53 | 2/20 | 26/55 |
| External validation set (n=42) | 0.83 | 0.95 | 0.52 | 1/21 | 10/21 |

*AUC: area under the curve

**Reference**

Tellez D et al (2019) Quantifying the effects of data augmentation and stain color normalization in convolutional neural networks for computational pathology. Med Image Anal 58:101544

A Bándi P et al (2019) Resolution-agnostic tissue segmentation in whole-slide histopathology images with convolutional neural networks. PeerJ 7:e8242

de Bel T et al (2019) Stain-transforming cycle-consistent generative adversarial networks for improved segmentation of renal histopathology. In: Proceedings of the 2nd International Conference on Medical Imaging with Deep Learning; Proceedings of Machine Learning Research

Radovic M et al (2017) Minimum redundancy maximum relevance feature selection approach for temporal gene expression data. BMC Bioinformatics 18(1):9
