# Supplementary material for: Artificial intelligence to detect MYC translocation in slides of diffuse large B-cell lymphoma
Source: Virchows Arch. 2020 Sep 26;479(3):617–21. doi: 10.1007/s00428-020-02931-4 (PMC8448690; doi:10.1007/s00428-020-02931-4)
Supplement: Supplementary file 2 — (PDF 559 kb) [file 428_2020_2931_MOESM2_ESM.pdf]

Table 1. Details of the individual DLBCL cases that were used for training, tuning and validation.

Set	*Case ID*	*MYC Fish*	*DL Result*	*High-grade*	*Centro-blastic*	*Immuno-blastic*	*Ana-plastic*	*Infla-mmation*	*Fibrosis*	*Site*	*IHC (Hans)*	*EBER*	*Hospital*
**Training**	74	**P**		x						stomach	GC	U	A
	76	**P**		x					x	skin	U	U	A
	78	**P**		x						testis	U	U	A
	79	**P**		x						oropharynx	non-GC	U	A
	111	**P**		x		x			x	soft tissue	CD10 N	N	A
	115	**P**		x						soft tissue	GC	U	A
	165	**P**		x		x				soft tissue	GC	U	D
	245	**P**		x						bone marrow	GC	U	A
	259	**P**		x						lymph node	U	N	A
	82	**P**			x					lymph node	GC	N	B
	97	**P**			x					lymph node	GC	N	A
	119	**P**			x				x	soft tissue	GC	U	A
	166	**P**			x					bone marrow	GC	N	A
	178	**P**			x					bone marrow	GC	N	A
	221	**P**			x					bone marrow	GC	U	A
	225	**P**			x					lymph node	CD10 N	N	A
	228	**P**			x					bone marrow	GC	N	A
	232	**P**			x					bone marrow	CD10 N	N	B
	108	**P**			x	x				stomach	non-GC	U	A
	164	**P**			x	x				bone	non-GC	N	A
	80	**P**				x	x			lymph node	non-GC	P	A
	117	**P**				x				lymph node	GC	U	B
	180	**P**				x				soft tissue	GC	U	A
	184	**P**				x				lymph node	GC	U	A
	102	**P**					x	x		lymph node	GC	N	A
	72	**P**		NE						lymph node	GC	N	C
	81	**P**		NE						adrenal	GC	P	A
	122	**P**		NE						bone	GC	U	A
	160	**P**		NE						bone	U	U	A
	169	**P**		NE						soft tissue	GC	U	A
	96	**P**		NE						lymph node	non-GC	U	B
	11	**N**		x						muscle	GC	N	A
	12	**N**		x						bone	CD10 N	U	A

Training	19	**N**	x			brain	GC	N	A
	28	**N**	x			breast	non-GC	N	A
	94	**N**	x			soft tissue	GC	U	B
	124	**N**	x			lymph node	GC	N	B
	192	**N**	x			liver	GC	U	A
	267	**N**	x			tonsil	CD10 N	N	A
	103	**N**		x		bone	GC	U	A
	1	**N**		x		lymph node	non-GC	N	A
	3	**N**		x		lymph node	GC	N	B
	20	**N**		x		brain	GC	N	A
	21	**N**		x	x	abdomen	GC	N	A
	26	**N**		x	x	soft tissue	non-GC	U	A
	30	**N**		x		maxillary sinus	non-GC	U	A
	32	**N**		x		oral soft tissue	GC	N	A
	36	**N**		x		brain	non-GC	U	A
	38	**N**		x		brain	non-GC	U	A
	41	**N**		x		retroperitoneum	GC	U	A
	42	**N**		x		stomach	GC	U	A
	52	**N**		x		lymph node	non-GC	N	A
	53	**N**		x		stomach	CD10 N	N	A
	56	**N**		x		nasopharynx	non-GC	N	A
	64	**N**		x		lymph node	non-GC	N	A
	67	**N**		x		soft tissue	non-GC	N	A
	71	**N**		x		brain	non-GC	N	A
	85	**N**		x		lymph node	GC	N	B
	91	**N**		x		lymph node	CD10 N	N	B
	93	**N**		x		soft tissue	non-GC	N	A
	95	**N**		x		soft tissue	U	N	D
	104	**N**		x		soft tissue	GC	U	A
	105	**N**		x	x	thyroid	GC	U	A

**Training**	107	**N**	x		lymph node	non-GC	N	A
	123	**N**	x		brain	CD10 N	N	A
	125	**N**	x		vagina	non-GC	N	A
	128	**N**	x		lymph node	GC	P	B
	130	**N**	x		intestine	non-GC	N	A
	133	**N**	x		testis	GC	N	B
	135	**N**	x		liver	GC	N	B
	138	**N**	x		testis	CD10 N	U	A
	163	**N**	x		pharynx	U	N	A
	170	**N**	x		adrenal	CD10 N	N	A
	171	**N**	x	x	brain	GC	U	A
	172	**N**	x		bone	CD10 N	U	A
	176	**N**	x	x	bone	CD10 N	U	A
	177	**N**	x	x	thyroid	GC	N	A
	183	**N**	x		lymph node	CD10 N	U	D
	185	**N**	x		lymph node	GC	U	D
	187	**N**	x		soft tissue	GC	U	A
	189	**N**	x		testis	GC	N	A
	190	**N**	x		lymph node	CD10 N	N	A
	194	**N**	x		lymph node	GC	U	D
	196	**N**	x	x	tonsil	GC	N	A
	197	**N**	x		lymph node	GC	N	A
	198	**N**	x		brain	GC	N	A
	295	**N**	x		soft tissue	GC	N	B
	204	**N**	x		brain	CD10 N	N	A
	206	**N**	x		breast	GC	N	D
	207	**N**	x		bone	GC	N	D
	209	**N**	x	x	brain	GC	N	A
	211	**N**	x	x	colon	CD10 N	N	A
	214	**N**	x		bone	GC	U	A

**Training**	215	**N**	x					lymph node	GC	U	B
	216	**N**	x				x	soft tissue	GC	N	A
	217	**N**	x				x	breast	GC	U	B
	223	**N**	x					soft tissue	GC	N	B
	230	**N**	x				x	soft tissue	GC	U	D
	234	**N**	x				x	bone	GC	U	A
	238	**N**	x					brain	non-GC	U	A
	242	**N**	x					bone	U	U	A
	243	**N**	x					lymph node	GC	U	A
	248	**N**	x					kidney	non-GC	N	A
	257	**N**	x					brain	GC	U	A
	265	**N**	x					lymph node	non-GC	N	A
	34	**N**	x	x		x	x	lymph node	non-GC	N	A
	55	**N**	x	x				lymph node	non-GC	U	A
	61	**N**	x	x				brain	GC	N	D
	90	**N**	x	x				lymph node	CD10 N	N	B
	92	**N**	x	x				bone	non-GC	U	A
	208	**N**	x	x				tong	CD10 N	N	A
	213	**N**	x	x				lung	CD10 N	U	A
	231	**N**	x	x				soft tissue	non-GC	U	A
	250	**N**	x	x				spleen	non-GC	N	A
	262	**N**	x	x				skin	GC	N	A
	134	**N**		x				lymph node	non-GC	N	A
	193	**N**		x				lymph node	GC	U	A
	266	**N**		x		x		lymph node	non-GC	N	A
	27	**N**			x	x		rectum	U	P	A
	62	**N**			x			lymph node	U	P	A
	87	**N**			x			bone	GC	U	A
	131	**N**			x		x	soft tissue	GC	N	A
	132	**N**			x			soft tissue	GC	N	A

	188	**N**				x		parotid	CD10 N	N	A
	218	**N**				x		lymph node	CD10 N	N	A
	255	**N**				x	x	mesentery	GC	U	A
	5	**N**	NE				x	soft tissue	GC	U	A
	7	**N**	NE				x	soft tissue	CD10 N	U	A
	39	**N**	NE					uterus	non-GC	N	A
	65	**N**	NE				x	intestine	U	P	A
	106	**N**	NE				x	bone	GC	U	A
	127	**N**	NE				x	bone	GC	U	A
	129	**N**	NE				x	peritoneum	GC	N	B
	168	**N**	NE				x	soft tissue	GC	U	A
	174	**N**	NE					U	GC	U	A
	199	**N**	NE					lymph node	CD10 N	U	D
	200	**N**	NE					brain	CD10 N	U	A
	205	**N**	NE				x	pancreas	CD10 N	N	A
	212	**N**	NE				x	lung	GC	U	B
	236	**N**	NE					soft tissue	non-GC	U	D
	253	**P**	x					ileum	GC	N	A
	83	**P**	x					breast	GC	N	B
	201	**P**		x				lymph node	GC	U	C
	99	**P**		x				soft tissue	GC	N	B
	118	**P**		x				muscle	GC	N	C
	73	**P**		x				lymph node	GC	U	C
**Tuning**	162	**P**			x			bone marrow	GC	N	A
	110	**P**			x			breast	non-GC	U	A
	252	**P**	NE					duodenal	GC	N	A
	202	**N**	x					bone marrow	GC	U	D
	9	**N**		x				tonsil	GC	U	A
	13	**N**		x				tong	non-GC	N	B
	43	**N**		x				lung	GC	U	D

**Tuning**	66	**N**			x				lung	non-GC	N	A
	88	**N**			x				colon	GC	N	B
	89	**N**			x				colon	GC	U	B
	136	**N**			x				vagina	GC	N	B
	167	**N**			x				bone	U	U	A
	210	**N**			x				lymph node	U	N	A
	229	**N**			x				tong	GC	N	A
	240	**N**			x				nasal mucosa	non-GC	N	A
	247	**N**			x				soft tissue	U	U	A
	264	**N**			x				stomach	GC	U	A
	2	**N**			x			x	lymph node	GC	N	A
	18	**N**				x		x	bone	GC	N	A
	40	**N**				x			brain	non-GC	U	A
	63	**N**				x			brain	non-GC	U	A
	258	**N**				x			brain	non-GC	N	A
	86	**N**					x		lymph node	CD10 N	U	B
	137	**N**					x		testis	non-GC	N	A
	233	**N**		NE					bronchus	U	N	A
**Internal validation**	100	**P**	P	x					mediastinum	GC	U	A
	109	**P**	P	x				x	soft tissue	GC	U	D
	113	**P**	P	x					lymph node	GC	N	B
	121	**P**	P	x					muscle	GC	N	B
	126	**P**	P	x				x	soft tissue	GC	N	D
	101	**P**	FN		x				lymph node	GC	U	D
	112	**P**	FN		x			x	lymph node	GC	N	A
	75	**P**	P		x				bone marrow	GC	N	B
	77	**P**	P		x				retroperitoneum	non-GC	N	B
	98	**P**	P		x				lymph node	GC	N	B
	220	**P**	P		x			x	epidural	GC	N	B
	222	**P**	P		x			x	lymph node	GC	N	B

**Internal validation**	235	**P**	P		x					lymph node	CD10 N	U	B
	237	**P**	P		x				x	omentum	GC	U	B
	256	**P**	P		x					skin	non-GC	N	B
	175	**P**	P			x				bone marrow	GC	U	D
	182	**P**	P			x				lung	GC	N	D
	116	**P**	P				x			salivary gland	GC	N	C
	114	**P**	P	NE					x	soft tissue	GC	N	B
	195	**P**	P	NE						liver	GC	U	D
	4	**N**	FP		x					thyroid	GC	N	B
	16	**N**	FP		x					lymph node	non-GC	N	A
	25	**N**	FP		x					bone	GC	U	D
	33	**N**	FP		x					tonsil	non-GC	N	D
	35	**N**	FP		x					spleen	GC	N	A
	37	**N**	FP		x					appendix	GC	N	D
	45	**N**	FP		x					brain	U	P	D
	46	**N**	FP		x				x	lung	non-GC	U	D
	48	**N**	FP		x					lymph node	non-GC	N	B
	69	**N**	FP		x				x	thyroid	GC	N	D
	70	**N**	FP		x					colon	U	N	D
	203	**N**	FP		x					skin	GC	U	B
	270	**N**	FP		x				x	soft tissue	GC	N	A
	224	**N**	FP		x	x				tonsil	non-GC	N	A
	22	**N**	FP			x				lymph node	GC	N	B
	50	**N**	FP			x				lymph node	non-GC	U	B
	249	**N**	FP			x				U	U	U	D
	173	**N**	FP			x				lymph node	GC	U	A
	57	**N**	FP			x	x			testis	GC	U	B
	17	**N**	FP				x			brain	non-GC	N	B
	23	**N**	FP				x			lymph node	non-GC	N	B
	31	**N**	FP				x	x	x	mediast	GC	U	A

**Internal validation**	49	**N**	FP				x		x	lung	U	N	B
	271	**N**	FP				x	x		lymph node	non-GC	P	B
	51	**N**	FP	NE						breast	GC	U	B
	244	**N**	FP	NE					x	retroperitoneal	GC	U	B
	14	**N**	N	x						lymph node	GC	U	B
	246	**N**	N	x						liver	GC	U	B
	251	**N**	N	x						soft tissue	GC	N	B
	6	**N**	N		x					lymph node	non-GC	N	B
	10	**N**	N		x				x	mesentery	GC	U	A
	24	**N**	N		x					subcutis	GC	N	B
	29	**N**	N		x					tong	U	N	B
	47	**N**	N		x					brain	non-GC	N	A
	58	**N**	N		x					lung	non-GC	N	B
	60	**N**	N		x					lymph node	GC	N	B
	68	**N**	N		x					soft tissue	GC	N	B
	226	**N**	N		x				x	bone	GC	U	A
	239	**N**	N		x			x	x	lymph node	non-GC	N	D
	241	**N**	N		x					pancreas	non-GC	U	A
	272	**N**	N		x					abdomen	GC	N	B
	254	**N**	N		x					parotid	U	N	B
	260	**N**	N		x					lymph node	non-GC	N	B
	261	**N**	N		x				x	retroperitoneum	non-GC	N	B
	268	**N**	N		x			x		jejunum	CD10 N	N	B
	186	**N**	N		x					mesentery	GC	U	D
	269	**N**	N		x	x				ileum	non-GC	U	A
	8	**N**	N				x			lymph node	U	P	A
	15	**N**	N				X			bone	CD10 N	N	A
	44	**N**	N				x			lymph node	non-GC	N	A
	54	**N**	N				x			retroperitoneum	GC	N	B
	59	**N**	N				x			lymph node	GC	N	B

	161	**N**	N				x			retroperitoneum	U	U	D
	191	**N**	N				x	x		lymph node	non-GC	P	A
External validation	139	P	P	x						lymph node	GC	U	C
	140	P	P	x						pelvis	GC	U	C
	149	P	P	x						lymph node	GC	N	C
	152	P	P	x						soft tissue	GC	N	C
	159	P	P	x						soft tissue	GC	N	C
	147	P	FN		x					lymph node	CD10 N	U	C
	141	P	P		x					duodenum	GC	P	C
	142	P	P		x				x	lymph node	GC	U	C
	143	P	P		x					small bowel	GC	N	C
	144	P	P		x				x	small bowel	GC	N	C
	148	P	P		x					Soft tissue	GC	U	C
	153	P	P		x			x		lymph node	GC	N	C
	155	P	P		x				x	lymph node	GC	N	C
	157	P	P		x	x				lymph node	GC	N	C
	150	P	P			x				bone	CD10 N	N	C
	156	P	P			x				Soft tissue	GC	N	C
	154	P	P				x			peritoneum	GC	N	C
	145	P	P	NE						lymph node	GC	U	C
	146	P	P	NE						lymph node	GC	N	C
	151	P	P	NE						lymph node	GC	N	C
	158	P	P	NE						lung	U	N	C
	286	N	FP	x						lymph node	GC	N	C
	288	N	FP	x						lymph node	GC	N	C
	276	N	FP		x					lymph node	non-GC	N	C
	278	N	FP		x					lymph node	U	N	C
	283	N	FP		x				x	liver	GC	U	C
	291	N	FP		x					lymph node	non-GC	N	C
	293	N	FP		x					lymph node	non-GC	N	C

External validation	294	N	FP		x					lymph node	non-GC	N	C
	289	N	FP		x	x				tonsil	GC	N	C
	277	N	FP				x		x	lymph node	GC	N	C
	274	N	N		x					nasal cavity	GC	N	C
	275	N	N		x					soft tissue	GC	N	C
	279	N	N		x					tonsil	non-GC	N	C
	282	N	N		x					mediastinum	non-GC	N	C
	284	N	N		x					soft tissue	GC	U	C
	285	N	N		x					tonsil	GC	N	C
	287	N	N		x					mediastinum	non-GC	U	C
	292	N	N		x					lymph node	non-GC	U	C
	290	N	N		x			x		lymph node	non-GC	N	C
	280	N	N				x		x	lymph node	GC	N	C
	281	N	N	NE					x	lymph node	GC	N	C

* where: DL- deep learning; FN- false negative; FP- false positive; GC- germinal center; N-negative; NE- not evaluable; Non-GB- non- germinal center; P-positive; U-unknown; Hospitals: A-Radboud, B-CWZ, C- Rijnstate, D-other.
